# Endophthalmitis: controlling infection before and after cataract surgery

**Published:** 2008-03

**Authors:** Nuwan Niyadurupola, Nick Astbury

**Affiliations:** Specialist Registrar in Ophthalmology, Department of Ophthalmology, Norfolk and Norwich University Hospital NHS Trust, Colney Lane, Norwich NR4 7UY, UK. Email: nuwan.niya@doctors.org.uk; Consultant Ophthalmic Surgeon, Norfolk and Norwich University Hospital NHS Trust.

**Figure:**
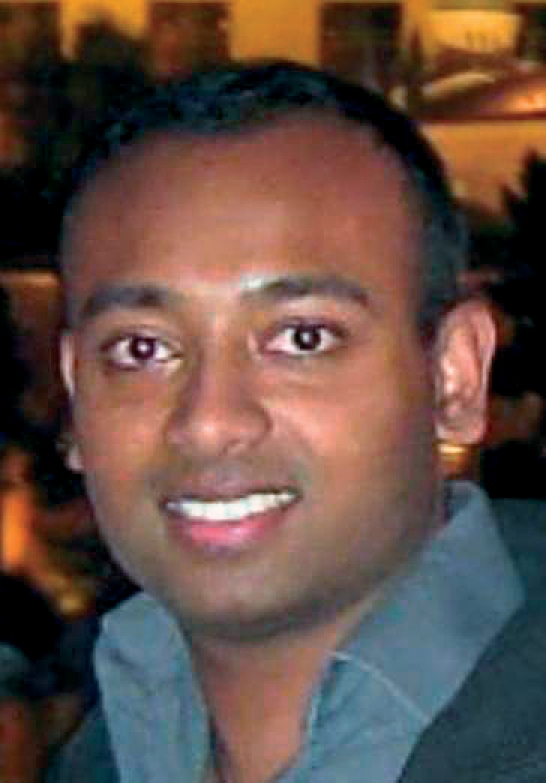


**Figure:**
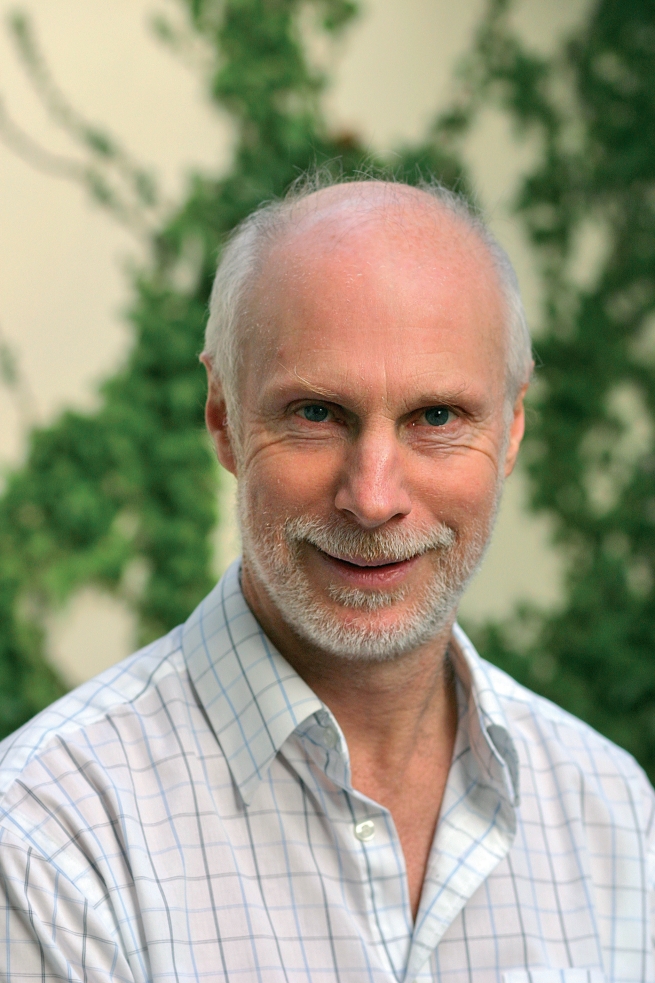


Endophthalmitis is a rare, but serious, postoperative complication of cataract surgery. It can have a devastating consequence on a patient's vision: some patients may lose all light perception.

The incidence of endophthalmitis has been reported to be between 0.13% and 0.7%.^1^ The primary source of this intraocular infection is considered to be bacteria from the patient's ocular surface (cornea, conjunctiva) or adnexa (lacrimal glands, eyelids, and extraocular muscles).^2^ The bacteria most frequently isolated are gram-positive coagulase-negative cocci (mainly *Staphylococcus epidermidis*) which account for 70% of culture-positive cases.^2^ *Staphylococcus aureu*s is isolated in 10% of culture-positive cases, *Streptococcus* species in 9%, *Enterococcus* species in 2%, and other gram-positive species in 3% of cases.^1^ Gram-negative bacteria account for just 6% of culture-positive cases; however, an infection with these bacteria, particularly with *Pseudomonas aeruginosa*, can lead to a devastating visual outcome.^1^^,^^3^

## Preoperative risk factors

Conditions that **increase the presence of bacteria on the ocular surface** are risk factors for the development of endophthalmitis.^1^ These conditions include: blepharitis, conjunctivitis, cannuliculitis, lacrimal duct obstruction, contact lens wear, and an ocular prosthesis in the fellow orbit.

**Eyelid abnormalities**, particularly the presence of entropion, also increase the risk of endophthalmitis. The correction or treatment of these risk factors prior to cataract surgery is desirable to reduce the risk of infection.

**Figure:**
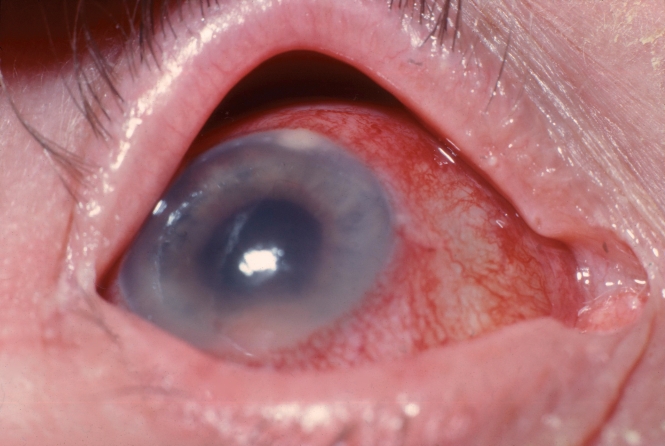
**Endophthalmitis**

**Recent immunosuppressive treatment** and a history of immunosuppression have also been shown to be significant risk factors for endophthalmitis.^4^

## Preparation of the patient

**Figure 1:**
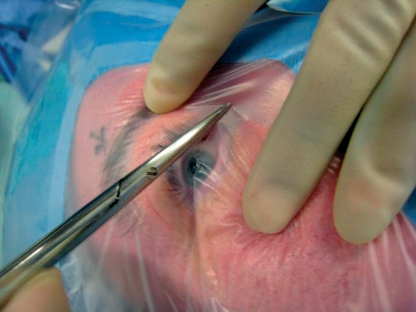
***Preparation of the drape covering the eyelid and lashes prior to cataract surgery***

The meticulous preparation of the patient for cataract surgery is possibly the most important factor in reducing the risk of endophthalmitis. It has been found that the **instillation of topical 5% povidone-iodine** (Betadine) into the conjunctival sac prior to surgery significantly reduces the risk of endophthalmitis; this has become accepted preoperative practice.^2^^,^^4^ The antimicrobial effect of povidone-iodine occurs within one minute of irrigation; it kills 96.7% of bacteria and lasts for at least one hour.^5^ Povidoneiodine appears to be more effective in reducing infection than preoperative antibiotics.^1^^,^^2^ As the bacteria responsible for endophthalmitis most commonly originate from the patient's eyelids, **careful draping of the eyelid and lashes** (Figure 1) is important in reducing the presence of bacteria in the surgical field, which in turn reduces the risk of endophthalmitis.^1^^,^^2^ The practice of trimming lashes is not recommended: it does not reduce periocular bacterial flora and does not reduce the risk of endophthalmitis.^5^

## Preparation of the surgeon

Proper **hand washing** (see page 17), followed by the use of **sterile gloves and gowns** during surgery, is accepted practice. However, there has been considerable discussion about the use of surgical masks. During a study in which culture plates were placed in the operative field, the wearing of surgical face masks was shown to significantly reduce bacterial cell counts.^6^ However, other studies have found that the use of face masks produces no reduction in airborne bacteria in theatre and no reduction in wound infection rates in general surgery.^6^ Other arguments for not wearing facemasks include: face masks increase condensation on operating microscopes, which may impair the surgeon's view; they may possibly cause rubbing off of facial skin squames into the operative field; and they impair communication.^6^ However, a recent case-control study showed that the use of face masks by the surgeon and the scrub nurse significantly reduced the risk of endophthalmitis (p<0.001).^4^ In conclusion, given the devastating consequences of endophthalmitis, the wearing of face masks is recommended.^7^

**Note:** Facemasks must be worn correctly; they must cover the nose, mouth, and chin completely and must never hang around the neck.

## Surgical technique and intraoperative factors

### Incisions

The clear corneal incisions commonly used for phacoemulsification are associated with a significantly increased risk of endophthalmitis, compared to **scleral tunnel incisions**.^2^^,^^8^ This may relate to differences in wound healing and potential wound leaks. The incidence of a flat anterior chamber is higher with clear corneal incisions than with scleral tunnel incisions.^2^ Temporal clear corneal incisions take longer to heal than scleral tunnel incisions and are also prone to fish-mouthing and trauma, thereby allowing access of bacteria into the eye.^2^

The construction of a **watertight corneal incision** is important in reducing the risk of intraocular infection. Straight-in and two-plane clear corneal incisions can leak; **threeplane incisions are better**.^2^ Starting the incision in the **vascular region of the limbus** results in an increased fibroblastic response, which may promote healing.^2^ Longer corneal incisions, in comparison to the incision width, are more stable than shorter length incisions and hence may reduce wound leaks and the risk of endophthalmitis.^2^^,^^9^

### Complications

Surgical complications, in particular a torn posterior lens capsule, can significantly increase the risk of endophthalmitis.^4^^,^^8^ This is backed up by animal studies. In one study on monkey eyes, the posterior capsule was shown to have a barrier effect against the development of endophthalmitis following the inoculation of bacteria into the anterior chamber.^4^ In another study, bacteria injected into the vitreous of rabbit eyes more readily caused endophthalmitis than bacteria injected into the anterior chamber.^4^ The association of endophthalmitis with surgical complications may explain the finding that cases of endophthalmitis are more prevalent when senior surgeons operate, as these surgeons may take on more technically difficult cases.^4^^,^^8^

### Intraocular lenses

The choice of intraocular lens (IOL) can affect the risk of endophthalmitis. The use of IOLs with silicone optics is associated with an increased risk of endophthalmitis, compared with that of IOLs with **acrylic optics**. It is unlikely that this is due to the hydrophobic nature of silicone, since a comparison of hydrophobic and hydrophilic lenses showed no difference in the rates of endophthalmitis.

The difference is more likely explained by the interaction of biofilms with the surface of the lens.^8^ The material used for the IOL haptic and the type of IOL (multipiece or one-piece) did not seem to affect the incidence of endophthalmitis.^8^ The use of injectable IOLs has been linked with a reduced risk of endophthalmitis, possibly as a result of the IOL not coming in contact with the tear film. However, there is often a strong correlation between the type of IOL insertion (forceps or injector) and the site of incision (scleral tunnel or clear corneal); it is considered that it is the site of incision that is the more important risk factor.^8^

### Antibiotics

There is little evidence that using antibiotics in irrigating fluid during surgery can reduce the risk of endophthalmitis. Vancomycin is the antibiotic most commonly used in irrigating fluid, due to its activity against gram-positive bacteria. However, the half-life of vancomycin in the anterior chamber is less than two hours and, for the most common gram-positive bacteria, it does not achieve concentrations in the anterior chamber above MIC90 (the concentration of the antibiotic at which 90% of bacteria are destroyed).^1^^,^^2^ Concerns about the emerging resistance to vancomycin, coupled with the lack of protective effect against endophthalmitis of antibiotics used in irrigating fluid, has led to the recommendation that vancomycin should not be used intraoperatively.^2^^,^^4^

In contrast, the **intracameral injection of the antibiotic cefuroxime (1 mg in 0.1 ml normal saline) at the conclusion of cataract surgery** has caused a reduction in the number of cases of endophthalmitis. The European Society of Cataract and Refractive Surgeons (ESCRS) multicentre study was stopped early when it was found that the absence of cefuroxime administration at the end of cataract surgery was associated with a five-to six-fold increase in the risk of endophthalmitis^8^ (see page 11).

Cefuroxime may also be protective against endophthalmitis when it is given as a **subconjunctival injection** at the end of surgery. Therapeutic levels of cefuroxime in the anterior chamber are achieved after 12 to 24 minutes following subconjunctival injection and levels continue to rise beyond two hours.^4^ There is some evidence for this: other subconjunctival antibiotics given at the conclusion of cataract surgery have been found to reduce the risk of endophthalmitis.^4^

## Postoperative treatment and follow-up

There is insufficient data on the effectiveness of the postoperative use of topical antibiotics in reducing rates of endophthalmitis, although this is a widespread pratice amongst surgeons.^1^ Following uncomplicated cataract surgery, the routine review of patients on the first postoperative day is not necessary, due to the low rate of sight-threatening complications.^10^ However, a review on the first day is probably recommended when patients have had complicated cataract surgery, surgery on an eye with co-existing disease (such as uveitis or glaucoma), or surgery performed on an only eye and when patients do not have ready access to eye services.^10^

## Summary

Multiple factors can lead to endophthalmitis. The source of the bacteria is considered to be from the patient's own ocular surface or adnexa. For this reason, simple measures in the preparation of the patient have a dramatic effect on the reduction of endophthalmitis rates, in particular the instillation of povidone-iodine and careful draping to isolate the lid and lashes. The use of antibiotics at the conclusion of surgery, especially intracameral or subconjunctival cefuroxime, is also recommended.

**Tips for preventing endophthalmitis**

- Instil povidone-iodine 5% eye drops prior to surgery.
- Carefully drape the eyelid and lashes prior to surgery.
- Use sterile gloves, gowns, and face masks.
- Construct watertight incisions, preferably three-plane.
- Manage complications (e.g. capsular rupture) effectively.
- Acrylic optics are better than silicone.
- Inject intracameral cefuroxime postoperatively (1 mg in 0.1 ml normal saline).

**Protocol for treating endophthalmitis**

- Admit the patient, stop antibiotics, and prepare for theatre.
- Perform a vitreous tap with or without capsulectomy.
- Give an intravitreal injection of vancomycin 2 mg and cefuroxime (or ceftazidime) 2 mg (or 0.5 mg amikacin if the patient is allergic to penicillin).
- Give a subconjunctival injection of vancomycin 50 mg and cefuroxime (or ceftazidime) 125 mg (or amikacin 50 mg if the patient is allergic to penicillin).
- Send the vitreous sample for microscopy and culture.
- Monitor the pain experienced by the patient. A reduction in pain suggests bacterial kill.
- Start instilling vancomycin 5% and ceftazidime 5% eyedrops hourly.
- If you cannot see the posterior segment, do an ultrasound B-scan, if this is available.
- If there is no improvement within 24 hours, consider repeating the vitreous sample and the antibiotic injections.
- Consider topical or systemic steroids if you are confident the infection is under control (i.e. pain is diminishing, fibrin is contracting, hypopyon is decreasing).
- Taper the treatment according to the patient's response and culture results.
- Keep the patient informed of progress.

**Note:** Vancomycin and cefuroxime (or ceftazidime) must not be mixed in the same syringe – draw up in separate syringes.

Reproduced by kind permission of The Royal College of Ophthalmologists
